# Our Congested Asylums

**Published:** 1910-01

**Authors:** 


### EDITORIAL DEPARTMENT.

### OUR CONGESTED ASYLUMS.

The insane are increasing in Texas faster than the State can make preparations to care for them. About every four years costly additions have to be made to each of the four asylums: meantime there are perhaps a thousand in jail and in county poorhouses. It is a burning disgrace to our civilization. For the most part the increase is due to alcohol, either directly or by inheritance. As it seems to be impossible to eradicate the cause, there is but one rational thing to do. Sterilize the males by vasectomy, as they do in Indiana, Oregon and California; prevent the propagation of a race of lunatics. There is now one lunatic to about every 500 people, and the ratio of increase is out of all proportion to the increase of population. I subjoin Dr. Preston's remarks on this subject, taken from his report as Superintendent of the Austin branch of the Texas State Insane Asylums. The cost to the State for the care of the five thousand insane last year was about three-quarters of a million dollars; to be exact $707,000, yearly average for five years. Dr. Preston says:

### DEFECTIVE POPULATION OF THE STATE.

It seems strange that in this so-called most enlightened age of the world's history that our present civilization is burdened with a defective population of one in 300, and that no remedy is at hand to stay its progress or lessen its weight. In no era in the world's history, so far as we have any knowledge, has such an effort been made as is made today to provide for such a defective population in numbers or condition. Each country, and each State in each country, seems to have for 100 years vied with one another in the magnificence of its buildings for the accommodation of its most defective class of inhabitants. Medical science and skill have been amply provided for the amelioration of the sad condition of these sorely afflicted people, and every other science has been ransacked in the hope of relief, and yet the progress of the malady goes on unabated and unchecked. We have spent our whole time in trying to remedy the evil as it exists, to cure the disease, instead of devoting at least a part of our time in staying the evil by removing the cause. It would take 100,000 men to stop a steam engine as it rolls swiftly along its track, yet one man with one hand can stop it almost instantly by simply turning off the steam and applying the brakes. He removes the cause and the engine stops. If we could only remove the cause the disease, insanity, would cease to exist. Can we do it? Not without the aid and concerted action of the whole people.

The remote cause of insanity, epilepsy, idiocy and much of criminality is an unstable nervous organization, due in a large measure to heredity. The immediate or exciting causes may be excesses along any line of life, embracing the abuse of alcohol and other drugs, venereal excesses—natural or unnatural—overwork or overstudy—straining alike the brain and the muscles, excessive religious excitement, excessive worry—entailing loss of sleep, venereal or other diseases and poor environment. By proper education and training many of the exciting causes can be removed. By the prevention of the procreation of defectives with hereditary predisposition either by enacting laws restraining marriage or by proper operations the hereditary causes of insanity can be in a great measure eliminated, and this can be done without injuring the health of the individual, and would add much to the health and stamina of future generations, and young people of today would live to witness the results of a few common-sense remedies applied with skill.

This suggestion may be ahead of the times, but it is certain to come in due course of human events, or it may come to pass that the defectives will be in the majority and may turn themselves out and turn the so-called sane in.

On the ground that the State has a right to eradicate any evil that is destroying the health and welfare of its citizenship, either present or prospective, several of our great Western States and one Southern State have passed laws restricting the marriage of idiots, epileptics and confirmed criminals, and authorizing the sterilizing of several classes of degenerates.

In the brief history of this asylum three generations of the same family have been inmates of this institution. One woman who has been admitted as a patient to this institution every year or so from recurrent mania for twenty years has in the meantime while out of the asylum borne ten children. One of the children is married to a young man who was for several years addicted to the abuse of alcohol, and his father was an epileptic. Six residents of this aylum are cousins, their insanity being due remotely to hereditary transmission.

I mention these few cases out of many that could be cited merely to illustrate the folly of allowing such defectives the power to continue their species. [To relieve the congestion chronic cases are furloughed: go home and propagate their kind.—EDITOR.]

It is true that nature is always striving to repair injuries that man is ever inflicting upon himself, and yet, though she is the best of all doctors, there must eventually be a limit to her power. Although education, training and proper environment can do much to improve the race, still we can never expect a strong and sturdy people from a defective ancestry. So long as we allow degenerates to propagate their kind, so long will the best blood of the land be burdened with the task of eliminating the defects thus engendered. No man would think of breeding his good and poor animals promiscuously if he expected to raise high grade stock, and yet we have no law forbidding the consumptive, who has but six months to live, and those who are of unstable nervous organizations, erratic and without reason to mix their blood through marriage with any one of their choice, often entailing ill-health, short life and hereditary mental defects upon several generations to come.

